# Role of the Inflammatory Response of RAW 264.7 Cells in the Metastasis of Novel Cancer Stem-Like Cells

**DOI:** 10.3390/medicina57080778

**Published:** 2021-07-30

**Authors:** Chan-Yen Kuo, Tzu-Hsien Yang, Pei-Fang Tsai, Chun-Hsien Yu

**Affiliations:** 1 Department of Research, Taipei Tzu Chi Hospital, Buddhist Tzu Chi Medical Foundation, New Taipei City 23142, Taiwan; flyskyjean@gmail.com (T.-H.Y.); n0210080209130901@yahoo.com.tw (P.-F.T.); 2 Department of Pediatrics, Taipei Tzu Chi Hospital, Buddhist Tzu Chi Medical Foundation, Taipei 23142, Taiwan; 3 Department of Pediatrics, School of Medicine, Tzu Chi University, Hualien 97071, Taiwan

**Keywords:** M2-like macrophage, reactive oxygen species, endoplasmic reticulum, RAW 264.7 cells

## Abstract

*Background and objectives*: Tumor progression and the immune response are intricately linked. Additionally, the presence of macrophages in the microenvironment is essential for carcinogenesis, but regulation of the polarization of M1- and M2-like macrophages and their role in metastasis remain unclear. Based on previous studies, both reactive oxygen species (ROS) and the endoplasmic reticulum (ER) are emerging as key players in macrophage polarization. While it is known that cancers alter macrophage inflammatory responses to promote tumor progression, there is limited knowledge regarding how they affect the macrophage-dependent innate host defense. *Materials and methods*: We detected the levels of ROS, the ability of chemotaxis, the expressions of markers of M1-/M2-like macrophages in RAW264.7 in presence of T2- and T2C-conditioned medium. *Results*: The results of this study indicated that ROS levels were decreased in RAW 264.7 cells when cultured with T2C-conditioned medium, while there was an improvement in chemotaxis abilities. We also found that the M2-like macrophages were characterized by an elongated shape in RAW 264.7 cells cultured in T2C-conditioned medium, which had increased CD206 expression but decreased expression of CD86 and inducible nitric oxide synthase. Suppression of ER stress shifted polarized M1-like macrophages toward an M2-like phenotype in RAW 264.7 cells cultured in T2C-conditioned medium. *Conclusions*: Taken together, we conclude that the polarization of macrophages is associated with the alteration of cell shape, ROS accumulation, and ER stress.

## 1. Introduction

Hepatocellular carcinoma (HCC) is one of the most common aggressive malignancies, with poor prognosis and high mortality [1].

HCC is one of the most aggressive human cancers and the third leading cause of death worldwide. Despite the recent advances in diagnosis and treatment of HCC, it remains a highly lethal disease due to the recurrence of metastasis.

The poor outcome of patients with HCC is correlated with metastasis based on a unique immune response signature of the liver microenvironment [2,3]. Accumulating evidence has demonstrated that in many solid tumor microenvironments, tumor-associated macrophages (TAMs) play a key role in cancer development [4,5,6,7]. The findings of numerous, recent experimental studies indicate that the polarization of TAMs has an important role in tumorigenesis, including metastasis [8,9,10].

Macrophages are cells with high plasticity that can adapt their profile according to specific environmental stimuli. Two extremes of macrophage activation continuum have been designated: M1 and M2. The M1 phenotype can be induced by IFN-γ alone or combined with microbial products, such as lipopolysaccharide (LPS). M1 cells release pro-inflammatory cytokines, such as tumor necrosis factor alpha (TNF-α), interleukin (IL)-12, and IL-23. They express high levels of major histocompatibility complex molecules and inducible nitric oxide synthase (iNOS), and can be cytotoxic against neoplastic cells. M2 or “alternatively activated” macrophages are induced by TH-2 type cytokines, such as IL-4, IL-10, and IL-13 [5]. These cells release anti-inflammatory cytokines such as IL-10, express high levels of arginase and scavenger receptor A (including mannose receptor, CD206), and have poor antigen presenting capability but enhanced debris clearance ability, which is important for promotion of wound healing and angiogenesis. TAMs and M2 macrophages share tumor-promoting functions and surface markers [7]. Their presence is associated with poor prognosis and survival of patients with several types of cancer [8]. The current understanding is that tumor cells highjack macrophages and utilize their M2-associated properties to fuel tumor growth. Hence, studying macrophage polarization by tumor cells has been a heavily investigated research field in the last decade with two major points of investigation being (i) whether macrophage polarization can be reverted to e.g., an M1 phenotype, and (ii) how tumor cells are able to induce the M2 phenotype in TAMs and use them for their benefit.

Classically, M1-like macrophages secrete pro-inflammatory cytokines and have an antitumorigenic role upon activation by LPS, TLR ligands, or IFN-γ. On the other hand, M2-like macrophages secrete anti-inflammatory cytokines IL-10 and TGF-β and have a protumorigenic role when activated by IL-13 and IL-4. Importantly, TAMs display an M2-like phenotype and exhibit protumorigenic features [11,12].

Several clinical studies have shown that increased number of TAMs frequently correlate with angiogenesis, metastasis, and poor prognosis Nevertheless, there is a consensus view that macrophage polarization is strongly related to tumor stage, suggesting that a dynamic switching from M1 phenotype, during the early phases of chronic inflammation, to an M2-like one might occur in established tumors. Moreover, several studies observed a “mixed” phenotype-expressing TAM population in different established murine and human tumors. For instance, Sugai et al. observed increased levels of both IL-12 and IL-10 in monocytes from progressed gastric cancer patients with respect to healthy donors.

Pharmacological drugs, such as clodronate-encapsulated liposomes or aminobisphosphonates, which knock down macrophages in vivo have reduced angiogenesis and tumor progression in several experimental tumor models. Recently, Zhang et al. showed that TAMs played an important role in tumor progression during sorafenib therapy. Clodronate-encapsulated liposomes and zoledronic acid, which deplete the macrophage population, are promising drugs that enhance the antitumor effects of sorafenib when used in combination.

Additionally, Zhang et al. reported that TAMs in lung adenocarcinoma have an M2-polarized subtype and are associated with poor prognoses, perhaps resulting from accelerated lymphangiogenesis and lymph node metastasis [13,14], and there were similar findings in other solid tumors [15,16,17].

In our previous study, we successfully established novel orthotopic and metastasis models in immunocompetent rats, and a novel rat cancer stem cell (CSC)-like cell line, T2C, was identified, which is useful not only for the study of metastasis of HCC but also for preclinical cancer drug screening [18]; however, the action mechanism of T2C and RAW 264.7 cells in the microenvironment remains unclear. In this study, we speculate that the treatment of T2C with RAW 264.7-conditioned medium will induce cell metastasis/migration but decrease reactive oxygen species (ROS) production.

Cancer is associated with oxidative stress, mediated through ROS generated mainly by malignant cells, granulocytes, TAMs, and MDSCs into the tumor microenvironment (TME). The TME includes a large number of different immune cell types among which MDSCs, TAMs, and Tregs concurrently work to restrain the immune response to a tumor, allowing for greater tumor invasion, metastasis, and resistance to treatments. Oxidative stress in the tumor microenvironment promotes immune suppression. It can reduce the infiltration of lymphocytes and favor the recruitment and accumulation of regulatory T cells and M2 TAMs. Antigen presentation by dendritic cells is impaired and tumor infiltrating lymphocytes are dysfunctional. Myeloid-derived suppressor cells and tumor-associated neutrophils inhibit lymphocyte functions through ROS production.

In lung and breast cancer models, ROS were essential for TAMs to invade the tumor niche and to acquire a protumorigenic M2 phenotype. Another study demonstrated that high intracellular ROS supported a more invasive phenotype in TAMs isolated from melanomas, possibly due to ROS-dependent tumor necrosis factor α secretion. The authors of this study found that at least part of the intracellular oxidative stress was endogenously generated by TAMs from melanomas, which expressed elevated levels of several mitochondrial biogenesis and respiratory chain genes. Moreover, macrophage-derived ROS drove the recruitment of Tregs to the TME for exerting tumor progressive roles. Moreover, H_2_O_2_ production by macrophages has also been proven to sustain tumor progression in gastric cancer via modulation of miR-328-CD44 signaling.

Experiments using primary cells are of high importance, but recent developments in biomedical research calling for implementation of the 3-R principles (animal replacement, refinement, and reduction) require alternative models. Accordingly, the acquisition of the M2 phenotype by the murine macrophage cell line RAW264.7 upon coculture with T2 cancer cells was examined as a novel research tool for investigating tumor-promoting macrophages and elucidating the role of different markers of TAMs, in particular ROS and ER stress.

## 2. Materials and Methods

### 2.1. Reagents

Dulbecco’s modified Eagle medium/Nutrient Mixture F12 (DMEM/F12), CellROX^®^ oxidative stress reagents, and Pierce BCA Protein Assay kit were from Thermo Fisher Scientific (Waltham, MA, USA).

### 2.2. Antibodies

Antibodies for Western blot analyses and the final working dilutions were as follows: iNOS (1:1000, Cat#PA3-030A, Thermo Fischer Scientific, Waltham, MA, USA), CD86 (1:1000, Cat#A1199, ABclonal Technology, Wuhan, China), CD206 (1:2000, Cat#A11192, ABclonal Technology, Wuhan, China), β-actin (1:5000, Cat#ab8227, Abcam, Cambridge, MA, USA), CHOP (1:1000, Cat#A0854, ABclonal Technology, Wuhan, China), ATF6 (1:1000, Cat#A12570, ABclonal Technology, Wuhan, China), and ATF4 (1:300, Cat#ab23760 Abcam, Cambridge, MA, USA).

### 2.3. Cell Culture

T2 and T2C cells were cultured according to our previous study [18]. The murine monocyte/macrophage cell line RAW 264.7 cells were purchased from the Bioresource Collection and Research Center (BCRC), Taiwan and cultured according to our previous study [19]. RAW 264.7 cells were cultured in T2- or T2C-conditioned medium for 24–48 h.

### 2.4. Measurement of Intracellular ROS Generation

The measurement of intracellular ROS generation was examined with CellROX^®^ oxidative stress reagents, according to the manufacturer’s instructions and a previous study [19]. The relative level of ROS was analyzed using a Coulter Cytomic FC 500 (Beckman, CA, USA).

### 2.5. Western Blotting

Protein was extracted from cells and subjected to Western blotting according to our previous study [18]. The intensities of the reactive bands were analyzed using ImageJ.

### 2.6. Chemotaxis Assay

The ability of chemotaxis was measured according to our previous study [19].

### 2.7. Statistical Analysis

Data are expressed as means ± SD. Groups were compared via the Student’s *t*-test. *p* < 0.05 was considered to indicate statistically significant differences.

## 3. Results

### 3.1. Decrease in ROS Accumulation in Macrophage RAW 264.7 Cells Cultured with T2C-Conditioned Medium

**Figure 1:**
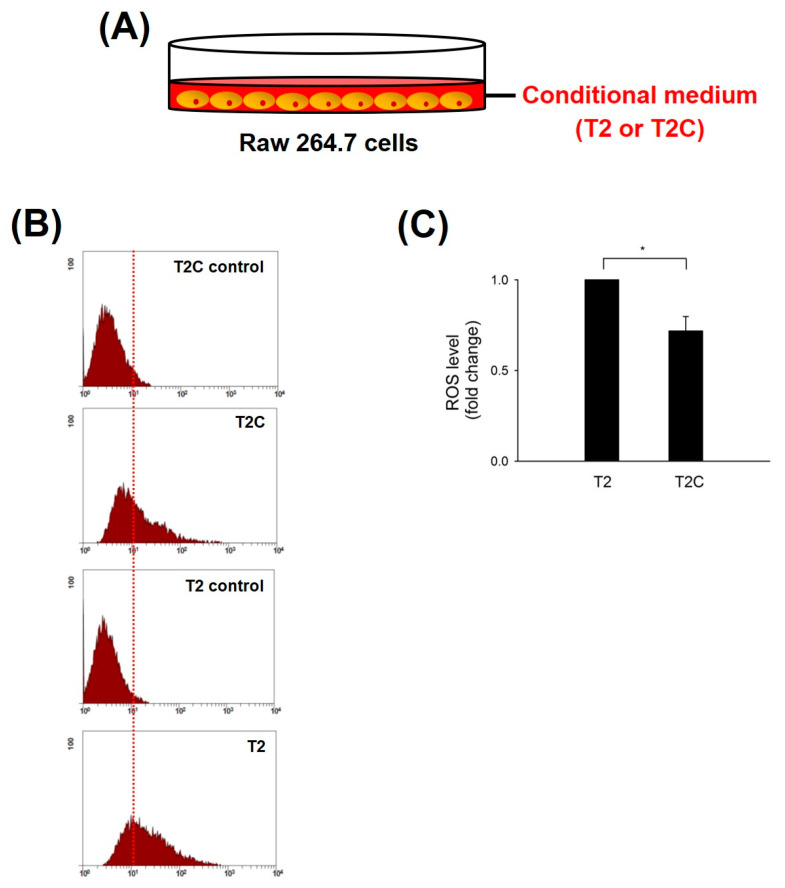
Effect of RAW 264.7 cells, cultured in T2- or T2C-conditioned medium, on ROS production. (**A**) Mode of RAW 264.7 cells cultured in T2- or T2C-conditioned medium. (**B**) After incubation, the levels of intracellular ROS were determined using DCF-DA, and (**C**) the fluorescence was detected using Coulter Cytomic FC 500. All data are presented as the mean ± SD. * *p* < 0.05 as compared with T2 group.

ROS produced in macrophages play a pivotal role in the tumor microenvironment (TME) of solid tumors during initiation, progression, and metastatic outgrowth of solid tumors [20]. Zhang et al. demonstrated that the inhibition of ROS blocked the activation and occurrence of M2-like macrophages and markedly suppressed tumorigenesis in mouse cancer models [21]. Thus, we studied ROS levels in RAW 264.7 cells treated with T2- and T2C-conditioned medium. The results show that ROS levels were significantly decreased in RAW 264.7 cells treated with T2C-conditioned medium (Figure 1).

### 3.2. T2C-Conditioned Medium Sensitizes and Improves Chemotaxis Abilities of Macrophage RAW 264.7 Cells

**Figure 2:**
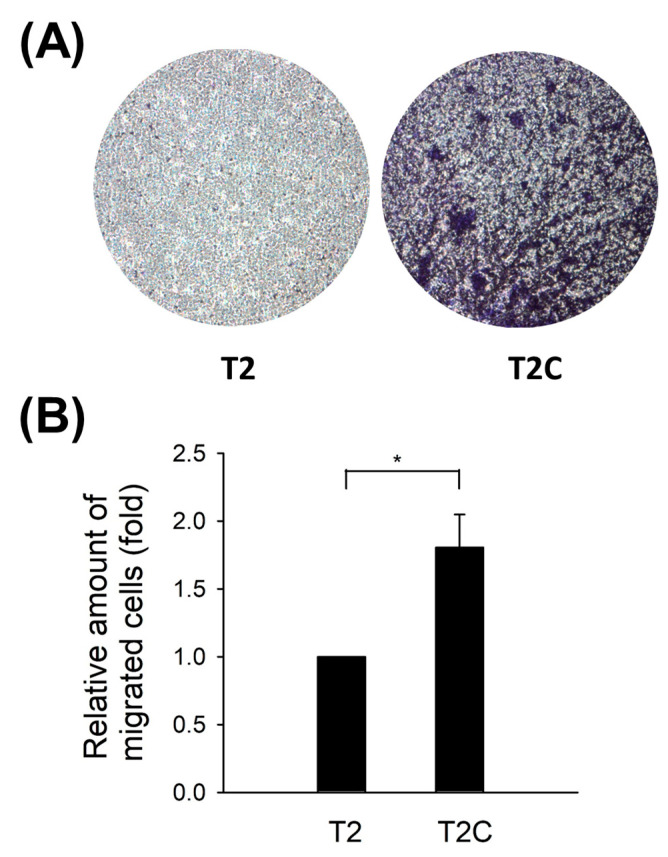
T2C-conditioned medium-improved chemotaxis abilities of RAW 264.7 cells. (**A**) Macroscopic observation of the transwell chamber. Briefly, T2- or T2C-conditioned medium was added into the lower well. At the same time, RAW 264.7 cells (1 × 10^5^) were added to the upper well and incubated for 24 h for chemotaxis assay. (**B**) Absorption measured at 570 nm. All data are presented as the mean ± SD. * *p* < 0.05 as compared with T2 group.

Qian and Pollard reported that macrophages accumulate at the metastatic site to promote target tissues as a destination for tumor cells and increase tumor cell extravasation, survival, and subsequent growth [22]. Moreover, macrophage infiltration has been considered a key contributor to metastasis [23,24]. As shown in Figure 2, an improvement in the chemotaxis abilities of RAW 264.7 cells after treatment with T2C-conditioned medium was determined (Figure 2).

### 3.3. Macrophage RAW 264.7 Cell Polarization to the M2-Like State Is Associated with Changes in Cell Shape in the T2C-Conditioned Medium

**Figure 3:**
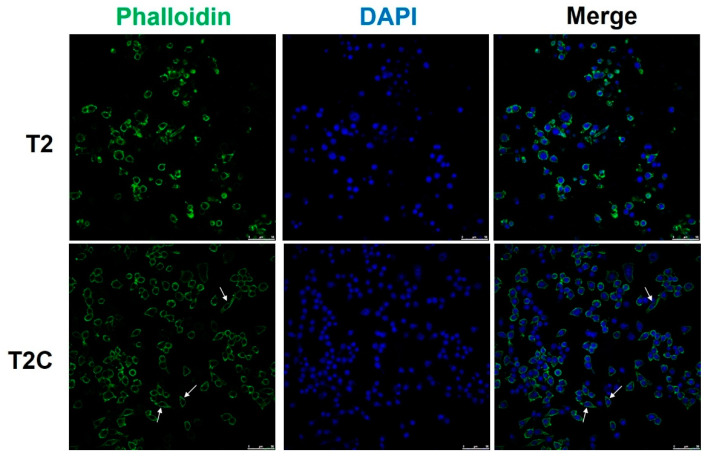
Polarization of macrophages toward an M2 phenotype is associated with an elongated cell shape in those treated with T2C-conditined medium. Fluorescence images of cell immunofluorescence staining for phalloidin (green) and DAPI nuclear counterstain (blue). Scale bar: 50 μm.

McWhorter et al. reported that cells take on a round, pancake-like shape via M1 polarization after being treated with IFN-γ and LPS, as well as a cellular elongation-like shape via M2 polarization after being treated with IL-4 and IL-13 [25]. Our phalloidin staining results indicated that an elongation-like shape results from cell growth in T2C-conditioned medium (Figure 3, arrows, lower panel), but a pancake-like shape resulted when using T2-conditioned medium (Figure 3, upper panel). Taken together, these data, as well as those of a previous study, suggest that cell shape is associated with the macrophage polarization state, and that M2 polarization is correlated with an increased degree of cell elongation [25].

### 3.4. Polarization to M2-like Phenotype in RAW 264.7 Cells Cultured in T2C-Conditioned Medium

**Figure 4:**
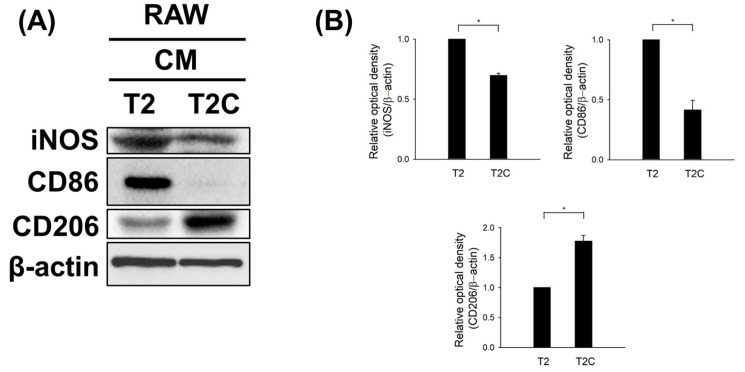
T2C-conditioned medium promoted the M1- to M2-like phenotype switch. (**A**) Cell lysates were subject to Western blot analysis with the indicated antibodies. β-Actin was used as an internal loading control. (**B**) The expression of indicated proteins was quantified using ImageJ. All data are presented as the mean ± SD. * *p* < 0.05.

To further examine whether T2- and T2C-conditioned media affect polarization, we characterized the expression of iNOS and CD86, typical M1-like macrophage markers, as well as CD206, a typical M2-like macrophage marker, using Western blotting. As shown in Figure 4, T2C-conditioned medium resulted in a marked decrease in the expression of iNOS and CD86 but an increase in the expression of CD206 (Figure 4). Thus, we suggest that T2C-conditioned medium stimulated polarization toward an M2-like phenotype.

### 3.5. ER Stress Affects Alternative M2-Like Polarization

**Figure 5:**
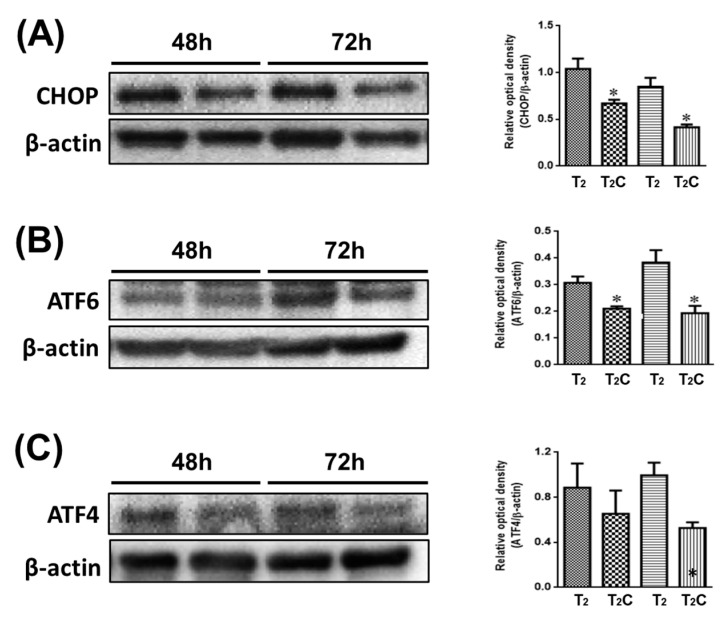
Effect of conditional medium (T2 and T2C) on polarization of RAW 264.7 cells involved in ER stress. Changes in the expression of (**A**) CHOP, (**B**) ATF6, and (**C**) ATF4. β-Actin was used as an internal loading control. Quantitative results show the level of specific proteins assessed by ImageJ. All data are presented as the mean ± SD (*n* = 3). * *p* < 0.05.

Minton demonstrated that the activation of ER stress has a role in suppressing the alternative M2-like polarization of macrophages [26]. As shown in Figure 5, it was observed via Western blotting that T2C-conditioned medium promoted a decrease in CHOP, ATF6, and ATF4 (Figure 5A–C). These results demonstrate that growth in T2C-conditioned medium stimulated the switch of macrophages to an M2-like phenotype via the inhibition of ER stress.

## 4. Discussion

The microenvironment of solid tumors is characterized by a reactive stroma with an abundance of inflammatory mediators and leukocytes, dysregulated vessels, and proteolytic enzymes. TAMs, major players in the connection between inflammation and cancer, summarize a number of functions (e.g., promotion of tumor cell proliferation and angiogenesis, incessant matrix turnover, repression of adaptive immunity), which ultimately have an important impact on disease progression [4,6]. Thus, together with other myeloid-related cells present at the tumor site (Tie2 macrophages and MDSCs), TAMs represent an attractive target of novel biological therapies of tumors [4,6]. However, the underlying mechanism of TAM polarization in metastasis still remains undefined. In the current study, we elucidated the role of ROS in mediating the macrophage polarization in HCC using RAW macrophage model.

Macrophages constitute an extremely heterogeneous population, which could be divided schematically into two main classes: M1 and M2. Blood monocytes differentiating in the presence of LPS/IFN-γ mature into M1-polarized cells (classically activated macrophages). They produce high levels of IL-12, IL-1, IL-23, TNF-, and CXCL10 and are characterized by cytotoxic activity against microorganisms and neoplastic cells, expression of high levels of ROI, and capability as APCs. On the other hand, when monocytes differentiate in the presence of IL-4, IL-13, IL-10, or corticosteroids, they mature into M2 macrophages (alternatively activated), which secrete IL-10, CCL17, CCL22, CCL18, IL-1ra, and IL-1R decoy. M2 cells are active workers of the host, promoting scavenging of debris, angiogenesis, remodeling, and repair of wounded/damaged tissues. Within the tumor mass, they exert the same functions favoring tumor promotion. In addition, M2 macrophages control the inflammatory response by down-regulating M1-mediated functions and adaptive immunity [4,6].

Macrophages are endogenous scavengers for dying cells in various pathological conditions, interaction between macrophages with compartments determines the phagocytic function of macrophages. Dying cells produce high levels of ROS, which are released into the extracellular area when the cellular membrane is degraded during cell death. Attachment of dying cells to macrophages requires intercellular communication in which ROS may play a role. On the other hand, extracellular and intracellular ROS may differentially control the phagocytosis process of macrophages by regulating the ability and capacity of macrophages in the uptake and degradation of dying compartments. In this regard, ROS play a critical regulatory role in determining the initiation and outcome of cellular phagocytosis [4,6].

ROS have been reported to play a pivotal role in the regulation of normal cellular process; however, deregulation of ROS may result in cancer formation [27,28]. Additionally, ROS mediate the tumor environment, including the process of metastasis, in a concentration-dependent manner [29]. Furthermore, high level ROS caused by several anticancer treatments have a role in the suppression of tumor metastasis via oxidative stress. However, metastasis occurs via DNA damage in tumor cells caused by sublethal levels of ROS [29]. Griess et al. suggested that M2-like macrophages have a lower level of ROS that is activated in a STAT3-dependent pathway, accelerating tumor cell metastasis [30]. Thus, a precise understanding of how ROS are generated and involved in tumor metastasis will help us to design better strategies to overcome such life-threatening events.

Involvement of ROS in regulating M1 responsible phagocytic activity and inflammatory response: Multiple pathways are involved in generating NADPH, followed by ROS production by NADPH oxidase. The high ROS level is mainly used to mediate the phagocytic activity of M1 macrophages. ROS serves as second messenger mediating the inflammatory response of M1 macrophages, primarily through MAPK and NF-κB as well as inflammasome activation [30].

Involvement of ROS in regulating M2 responsible inflammation resolving and wound healing activities: Multiple pathways are involved in reducing NADPH and its oxidase followed by reduced ROS generation. The low ROS level was accompanied by reduced inflammatory mediators; increased M2-regulated genes responsible for inflammation resolution; and increased disulfide protein degradation which enhanced wound healing effect of M2 [30].

Another study by Griess highlights a critical role of ROS in M2 macrophage function and implies that targeting the redox susceptibility of these macrophages could be a promising consideration for a more effective anticancer strategy. Scavenging ROS selectively inhibits M2 macrophage polarization and their protumorigenic function in part, via STAT3 suppression [30].

The ER is a dynamic membranous organelle that facilitates correct protein modification, folding, and maturation of transmembrane, secretory, and ER-resident proteins. Unfolded protein response (UPR) is an adaptive intracellular signaling pathway that responds to ER stress by attenuating global protein translation and degrading unfolded proteins. During monocyte differentiation by macrophage colony stimulating factor (M-CSF), the ER undergoes structural as well as functional reorganization to perform the new cell functions, leading to ER stress and upregulated UPR [31]

The UPR is a pro-survival mechanism that is activated and regulates cells by maintaining protein homeostasis via accumulation of unfolded, misfolded, and immature proteins in the ER; however, the UPR can also trigger cell death under unresolved levels of improper protein accumulation [32,33]. Moreover, aberrant activation of endoplasmic reticulum stress (ER stress) has been considered to have a major role in the regulation of tumor growth, invasion, metastasis, and its microenvironment, as well as in response to chemotherapy, targeted therapies, and immunotherapy [34,35]. The alleviation of ER stress prevents the otherwise increasing potential of cells to acquire or sustain stem-cell properties and metastatic capacity, thereby enhancing the malignancy of oral squamous cell carcinoma cells by increasing the population of cancer stem or stem-like cells [36]. Therefore, the UPR has a potential role in the regulation of the development of carcinogenesis via ER stress; however, due to the paradoxical outcomes of UPR activation as well as gaps in current knowledge, further studies are required to confirm this hypothesis [36].

The levels of ROS production have been reported to be correlated with not only tumor metastasis but also most disease processes [20]. Moderate ROS production in tumor cells can cause pathological conditions and promote tumor formation and progression via increases in the metabolic rate, DNA mutation, and angiogenesis, and excess ROS can be eliminated by increased activity of antioxidant pathways in redox-adapted cancer cells. On the other hand, ROS accumulated at a high level may also be able to inhibit carcinogenesis via triggering programed cell death [37,38]. The conflicting results indicate that either pro- or antitumorigenic ROS signaling pathways can be manipulated during the treatment of cancer [27]. This is likely, in part, due to elevated mTOR signaling in these mutant cells [39,40]. Moreover, it has been well demonstrated that the overwhelming activation of immune cells may be a major contributor to tissue damage during inflammatory diseases. Neutrophils represent a major cell population in the human innate immune system, which is the first line of defense against bacterial invasion [41]. However, neutrophils are regarded as destructive cells that secrete toxic ROS and proteolytic enzymes that destroy the surrounding tissue [42]. Accumulated neutrophils induce endothelial dysfunction and microcirculatory collapse in acute coronary syndrome and in a myocardial ischemia/reperfusion injury model [43]. Interestingly, Zhang et al. suggested that ROS play a critical role in the differentiation of alternatively activated macrophages and in the occurrence of TAMs [21]. Moreover, Wu et al. demonstrated that the exosome-mediated transfer of the functional CD11b/CD18 protein from TAMs to tumor cells may have the potency to boost the migratory potential of HCC cells, thus providing insights into the mechanism of tumor metastasis [44]. Overall, we expect that targeting ROS will represent a fruitful ground for future molecular anticancer strategies.

It is well known that cancer cells have higher intracellular ROS levels than their normal counterparts. It is also becoming clear that the TME is highly oxidized compared to their normal tissue counterparts. However, the role of the oxidative TME on cellular functions within the tumor remains relatively understudied. It has been shown that the oxidative TME contributes to immunosuppression in cancer, where increased extracellular ROS inhibited CD8^+^ T cell activation, while inducing Treg activation. Our data show that M2 or TAM-like macrophages have increased redox buffer, suggesting their high tolerance within the oxidative TME. Additionally, this study indicates ROS is a necessary secondary messenger for proper M2 polarization and function. Furthermore, addition of exogenous hydrogen peroxide promotes M2 polarization. Thus, it is reasonable to speculate that the oxidative TME may actively promote an immunosuppressive environment, via polarizing TAM to a M2-like phenotype, while also inhibiting T cell activation and increasing Tregs [45].

M2 macrophages have differential expression of ROS scavenging enzymes and ROS promotes IL-4 stimulated M2 polarization. Some key differences in the expression levels of ROS producing and scavenging enzymes were identified by Beyer et al. in M1 vs. M2 macrophages [45]. Their results show that M2 macrophages have increased expression of genes in the peroxiredoxin family, specifically PRDX1, PRDX3, and PRDX6, which may further contribute to the lower levels of ROS in M2 macrophages. Some of the largest changes in ROS-related genes were seen in the glutathione-S-transferase family, specifically lower GSTO1 and higher GSTP1, mGST2, and GSTT1, in M2 macrophages, which may lead to different glutathionylation patterns as a potential regulator of macrophage polarization and function [45].

Despite our knowledge that polarization of macrophages is essential in the progress of tumorigenesis, the molecular mechanisms controlling their phenotype and the interplay in M1- and M2-like phenotypes remain unclear. Liu et al. summarized that M1-like macrophages mainly rely on glycolysis and exhibit impairment of the tricarboxylic acid cycle and mitochondrial oxidative phosphorylation (OXPHOS), but M2-type macrophages are closely associated with OXPHOS-dependent pathways [46]. Furthermore, the abrogation of the metabolic ER sensor IRE1α promotes emergence in the M2-like population but decreases the M1 polarization of macrophages in vivo [47]. Zhao et al. proposed that the suppression of M1 polarization occurs via inhibition of the ER stress-associated IRE-1/XBP-1 signaling pathway and led to ameliorated LPS-induced lung injury [48]. Thus, these results indicate that the polarization of M2-like macrophage was a result of ER stress inhibition, which is in accordance with the findings of our study (Figure 5). However, there have been conflicting results showing that ER stress in macrophages was activated by palmitic acid, which induced its M1-phenotypic shift in the PERK–eIF2α-dependent pathway [49]. In a clinical study of diabetic patients, M2-like macrophages shifted toward M1-like macrophages via the suppression of ER stress/JNK activation and upregulation of CCR7 [50]. We propose that the conflicting role of ER stress in M1- and M2-like macrophage polarization is based on the molecular mechanism of ER stress activation, which is not simple, as it involves signaling pathways that have cellular autophagy, oxidative stress, and inflammatory responses [51]. Therefore, decoding how the ER stress pathway signals cellular and inflammatory responses and preventing them are major challenges for future research and will require definition before being utilized in rationales for drug design and application in anticancer formulations involving macrophage polarization.

## 5. Conclusions

**Figure 6:**
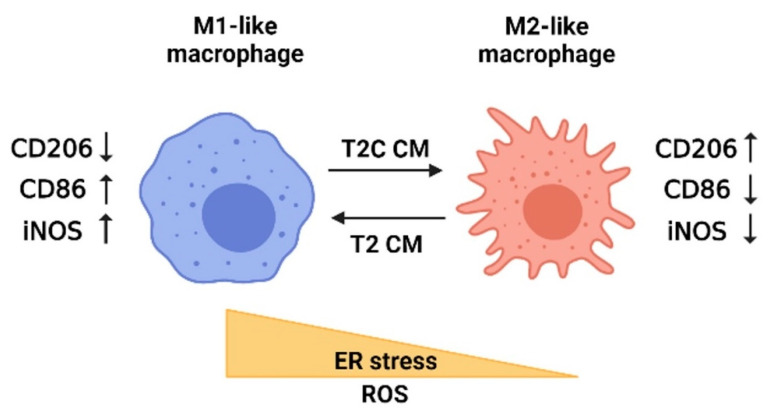
Model depicting the influence of ER stress and ROS on macrophage polarization based on the study results. The polarization of macrophages is mediated by ER stress and ROS. M2-like macrophages are detected after treatment with T2C-conditioned medium (T2C CM) and characterized by increased CD206 but decreased CD86 and iNOS. By contrast, M1-like macrophages are detected after treatment with T2-conditioned medium (T2 CM) and characterized by decreased CD206 but increased CD86 and iNOS. The figure was created using BioRender.com.

In this present study, we identified the critical role of the M2-like inflammatory response of RAW 264.7 cells in regulating the metastasis of cancer stem-like cells via ER stress (Figure 6); however, more evidence is required to further elucidate which signaling pathways are involved. Moreover, eradication of M2-like macrophages by targeting ER stress is of great interest in regard to the design of novel anticancer therapies for HCC.

This study would be a guide for further studies targeting ROS production in macrophages and can be used in all solid tumors where the TME is dominated by TAMs like pancreatic cancer, breast cancer, HCC. In patients with nonresectable or advanced HCC, the recent introduction of the molecular targeting agent, sorafenib, was reported to improve survival rates. Sorafenib is a multikinase inhibitor found to prolong survival significantly, at least by less than 3 months. This study can be used in combination to show the effects on similar drugs demonstrating the downregulation of macrophage polarization in HCC.

Thus, ROS-driven macrophage polarization is too difficult to become a putative drug target owing to the lack of specificity. This shortage is reflected in multiple levels, and pharmaceutical companies have to develop systems that particularly target macrophage ROS as well as its polarization. This includes not only drug target study but also development of a drug delivery system. Potential molecules targeting ROS signaling and macrophage reprogramming could be further enriched in order to discover the target treatment. More experimental investigations as well as clinical trials should be conducted to prove the hypothesis. Further studies on factors like differential expression of Nox2, Duox1, DuoxA1, Mn-SOD, catalase, Gpx1, and Cu/Zn-SOD in M1 and M2 macrophages, cytokine profile (IL-4), STAT4 expression, and similar studies need to be carried out.

## Data Availability

The original data used to support the findings of this study are available upon reasonable request.
